# Supplementary material for: lesSDRF is more: maximizing the value of proteomics data through streamlined metadata annotation
Source: Nat Commun. 2023 Oct 24;14:6743. doi: 10.1038/s41467-023-42543-5 (PMC10598006; doi:10.1038/s41467-023-42543-5)
Supplement: Supplementary file 1 — Supplementary information [file 41467_2023_42543_MOESM1_ESM.pdf]

Supplementary information

Text mining effort

To extract metadata from public data sets in the PRIDE database, we employed manual text mining using ontology terms obtained from the OLS (Ontology Lookup Service, https://www.ebi.ac.uk/ols/index). A group of seven 3rd year Biotechnology students selected a random sample of projects from PRIDE in the context of a general data reprocessing effort. This resulted in the annotation of 355 proteomics projects along with their accompanying research articles. Of these projects, 262 had associated open-access research articles in PubMed Central that were retained for further analysis. Moreover, we removed 21 protein cross-linking experiments that did not fit with the original objective of the reprocessing study, resulting in 241 projects for further metadata annotation (**Supplementary Figure 1).** Metadata categories included biological metadata (species and tissue/cell line) and technical metadata (protease, labeling technique, instrument, fragmentation type, number of missed cleavages, precursor ion mass tolerance, fragment ion mass tolerance, variable modifications, and fixed modifications). Following annotation, all students and supervisors conducted peer reviews to ensure accuracy and consistency of the annotated projects. A list of all the PRIDE data set identifiers (PXD) of the annotated projects is included as **Supplementary Data 1**, the results of the text mining were compiled into a final csv file, which is available as **Supplementary Data 2** and **Supplementary Table 1** contains an overview summary of the different experiment types, labeling methods and sample types identified.

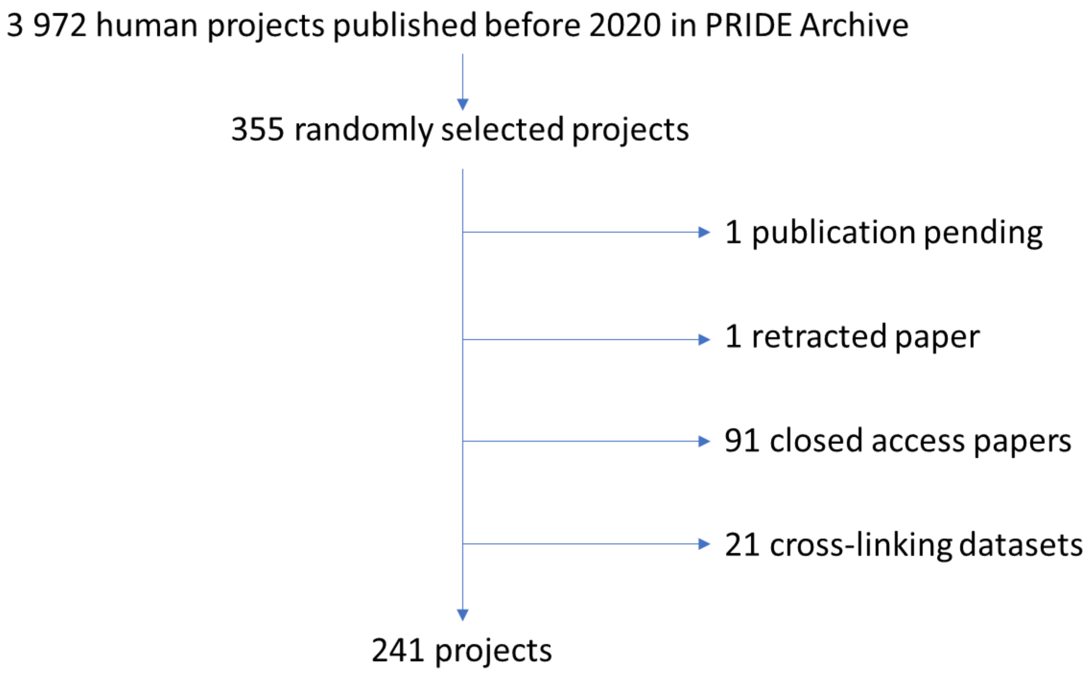

Supplemental figure 1. *The figure outlines the project selection process, starting with 3,972 human data projects published before 2020. A random subset of 355 was initially selected. After excluding projects with pending or retracted publications, closed-access papers, and those focused on cross-linking, 241 projects remained for the study.*

Development of the lesSDRF web application

The *lesSDRF* web application was built using Streamlit version 1.19.0 and Python version 3.9.13. The following ontologies/CVs were downloaded: PRIDE CV (version 2022-11-17), PSI-MS (version 2022-09-26) and NCBITaxon (version 2022-08-18) in obo format, CL (version 2022-12-25) and HANCESTRO (version 2.6) in OWL format, and EFO (version 3.49.0) in JSON format. Data from the Unimod database for protein modifications was also copied in csv format from their website. Regular updates of these ontologies are scheduled. The downloaded ontologies were stored and parsed into three types of JSON files. First, all the elements from the ontology were stored into a list, which was then stored as an "all_elements.json" file. Second, the ontology was stored as a nested dictionary reflecting the ontology tree. Lastly, the ontology was stored as a tree structure compatible with the streamlit_tree_select module from https://github.com/Schluca/streamlit_tree_select which is used to generate the ontology tree visualization and is referred to as a "nodes.json" file. All JSON files were gzipped to reduce space. The home page of the application used the SDRF templates based on species from https://github.com/bigbio/proteomics-sample-metadata/blob/master/sdrf-proteomics/README.adoc as a starting format of the SDRF. This format consists of all the *required* columns. Additional columns, which were selected based on the same GitHub page and personal experience, can then be added in the next stage. The editable data frames were generated using the streamlit AG grid module from https://github.com/PablocFonseca/streamlit-aggrid. All code used to create lesSDRF is available via https://github.com/compomics/lesSDRF.
Required packages and their versions are:
pronto== 2.5.3
streamlit==1.19.0
streamlit-aggrid==0.3.4.post3
streamlit-tree-select==0.0.5
jsonschema==4.17.0
zipp==3.10.0
openpyxl== 3.1.1
